# Geographical structures and the cholera epidemic in modern Japan: Fukushima prefecture in 1882 and 1895

**DOI:** 10.1186/1476-072X-6-25

**Published:** 2007-06-30

**Authors:** Chun-Lin Kuo, Hiromichi Fukui

**Affiliations:** 1 Center for Geographic Information Science, Research Center for Humanities and Social Sciences, Academia Sinica, Taipei, Taiwan; 2 Faculty of Policy Management, Keio University, Kanagawa, Japan

## Abstract

**Background:**

Disease diffusion patterns can provide clues for understanding geographical change. Fukushima, a rural prefecture in northeast Japan, was chosen for a case study of the late nineteenth century cholera epidemic that occurred in that country. Two volumes of *Cholera Ryu-ko Kiji (Cholera Epidemic Report)*, published by the prefectural government in 1882 and 1895, provide valuable records for analyzing and modelling diffusion. Text descriptions and numerical evidence culled from the reports were incorporated into a temporal-spatial study framework using geographic information system (GIS) and geo-statistical techniques.

**Results:**

Changes in diffusion patterns between 1882 and 1895 reflect improvements in the Fukushima transportation system and growth in social-economic networks. The data reveal different diffusion systems in separate regions in which residents of Fukushima and neighboring prefectures interacted. Our model also shows that an area in the prefecture's northern interior was dominated by a mix of diffusion processes (contagious and hierarchical), that the southern coastal region was affected by a contagious process, and that other infected areas experienced relocation diffusion.

**Conclusion:**

In addition to enhancing our understanding of epidemics, the spatial-temporal patterns of cholera diffusion offer opportunities for studying regional change in modern Japan. By highlighting the dynamics of regional reorganization, our findings can be used to better understand the formation of an urban hierarchy in late nineteenth century Japan.

## Background

Researchers from different disciplines are showing a growing interest in disease and its geographical effects, with studies focusing on the value of detecting spatial concentrations of disease, isolating processes that result in disease hot-spots, and analyzing the space-time dynamics of disease diffusion. A strong example of recent advancements in this area is [2] work on the geographical structures of international epidemics, resulting in models of how epidemic diffusions move through communities, regions and countries. The term *geographical structures *refers to the patterns and features of human-environment interactions in specific locations. In medical geography, studies of the geographical structures of disease emphasize diffusion and analyses of individual disease factors [8].

Regarding cholera, the most serious global epidemic in the nineteenth century, several research teams have gathered evidence showing that its diffusion was dominated by geographic factors (see, for example, [3-6]). Since diffusion primarily occurs via survivors who transport a disease from one location to another, diffusion routes represent community interactions and seaborne or overland transport between villages, towns, or regions. Geographic factors such as traffic systems, population density, and the presence of an urban hierarchy can spatially dominate disease diffusion. However, there is little empirical data supporting the idea that visualizing an epidemic's spatial-temporal patterns can assist in the framing of geographical structures, especially during periods of rapid change.

In this paper we present a case study of regional transition in a rural Japanese prefecture during the late nineteenth century. Our goal is to demonstrate the potential use of GIS-based methodology to explore both cholera diffusion dynamics and ways that regional changes are presented in historical epidemic records. We have three reasons for using cholera diffusion to measure geographic change: (a) the availability of detailed historical records that describe local sanitary and disease conditions during a period of national modernization; (b) the transmission characteristics of cholera and its uncontrolled spread in late nineteenth century Japan are suitable for modelling temporal and spatial change; and (c) a combination of the availability of fully developed GIS software for geo-statistical analyses and advancements in disease studies (e.g., [9,10]).

After introducing the data found in the two cholera reports and features of the Fukushima epidemic outbreaks, we describe how GIS-based methodology was used to model and analyze disease diffusion. Our results are presented as visual representations of disease patterns and identified diffusion systems prior to modelling diffusion processes. We present three major findings regarding regional transitions before offering our conclusion.

### Cholera Ryu-co Ki-ji

Following the Meiji Restoration, Japan endured a series of cholera outbreaks every 3–5 years from the 1870s to 1895. As part of a modern medical regulatory system established in 1876, several prefectural governments published *Cholera Ryu-co Ki-ji *(Cholera Epidemic Reports) following each outbreak. These documents are now being used to analyze specific epidemic outbreaks and changes in Japan's social-economic structure. Fukushima prefecture released two sets of *Cholera Ryu-co Ki-ji*, the first published by the police department in 1882 and the second by the prefecture's sanitary agency in 1895. Each report contains data on the number of patients, gender, occupation, age, symptoms, treatment, and how disease prevention laws were applied. The 1895 report is considered more accurate and complete, in part due to progress made in establishing disease recording and reporting systems over the preceding decade.

The contents of the Fukushima cholera reports can be divided into two categories. The first consists of numerical evidence such as the number of cases reported in infected villages. The beginning and ending dates of outbreaks in each village were clearly noted in these reports. The second category consists of textual accounts of diffusion routes, morbidity, and mortality. Also recorded were possible factors for the diffusion of cholera and measures taken to combat its spread.

### Features of the Fukushima Epidemic Outbreaks

**Figure 1:**
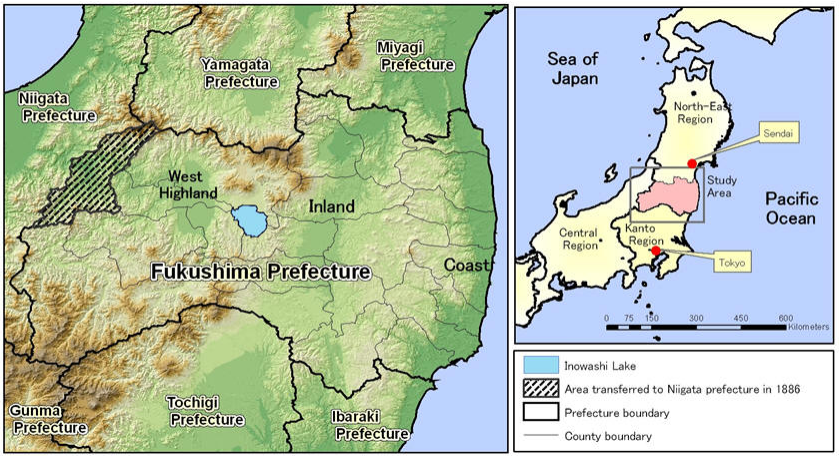
Study area: Fukushima prefecture in the late nineteenth century.

Established in 1876, Fukushima prefecture was at the time Japan's third largest prefecture in terms of area. Its position along the coast in northeast Japan made it an important link between the cities of Tokyo and Sendai (Fig. 1). In the late nineteenth century, several cholera outbreaks gradually spread from southern prefectures to northeast Japan [1]. Due to its location, Fukushima could not avoid being hit full-force by each outbreak. However, according to textual accounts in the two *Ryu-co Ki-ji*, there were substantial time lags between national and Fukushima outbreaks – it was one of the very last prefectures to feel the effects of the initial national diffusion. The reports also indicate that cholera entered the prefecture via a different route during each outbreak, that it suffered fewer cases than most prefectures, and that it rarely exported the disease.

**Figure 2:**
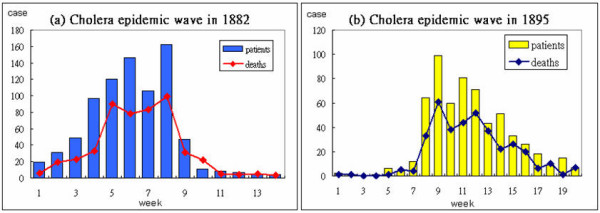
Graphs for comparing 1882 and 1895 epidemic waves.

Statistical data for the two outbreaks are summarized in Table 1. As shown, the 1895 epidemic started one month earlier than the 1882 epidemic, but end dates, mortality rates, and peak weeks are comparable. Figure 2 presents data on weekly cholera cases recorded during each outbreak. In 1882 the number of cases increased dramatically from week 1 to a peak of 160 cases in week 8; the number then steadily declined from week 9 to week 14. In 1895 the number of cases increased very slowly during the first six weeks, dramatically increased from week 7 to a peak in week 9, then slowly declined to its end in week 20. The major differences noted in the two data sets likely reflect structural changes enacted between the two outbreaks.

**Table 1:** Summary of 1882 and 1895 epidemic waves.

	*1882 Outbreak*	*1895 Outbreak*
**Start**	July 23	June 25
**End**	November 3	November 12
**Duration (weeks)**	14	20
**Deaths/Cases**	501/812	373/605
**Mortality**	61.70%	61.65 %

Peak	Week 8	Week 9

## Methods

Textual descriptions and numerical data culled from the two epidemic reports were used to trace diffusion routes. Thanks to the efforts of local doctors during that period we have clues for tracing the origins of these routes. Tables 2 and 3 present summaries of infected counties, first infected village in each county, disease entry and termination dates for each county, number of cases, and possible diffusion origins and routes for each of the two outbreaks. Due to the incomplete construction of sanitary systems in late nineteenth century Japan, some diffusion origin and route records are either fragmented or unconfirmed; we used GIS-based interpolation techniques to fill in the gaps.

**Table 2:** Motility and cholera diffusion routes in 1882.

**Outbreak Order**	**Infected County**	**First Infected Village**	**Entry Date**	**Termination Date**	**Cholera Cases**	**Diffusion Route**
1	Kitaaizu	Wakamatsu	July 25	Sep 14	6	-
2	Date	Ozeki, Ryogawa	Jul 26	Oct 23	254	Miyagi Pref.
3	Iwaki	Yotsukura	Aug 3	Sep 26	41	-
4	Yukikata	Minamiebi	Aug 6	Oct 5	55	Miyagi Pref.
5	Yuha	Tanomi	Aug 7	Oct 10	45	Iwaki County
6	Adachi	Kitasugita	Aug 8	Sep 26	33	-
7	Yama	Miyazu	Aug 9	Sep 7	3	Kawanuma
8	Iwamae	Shiota	Aug 9	Oct 14	64	-
9	HigashiKamahara	Koishitori	Aug 11	Sep 3	5	Niigata Pref.
10	Azumi	Kaidoshita	Aug 15	Sep 9	9	-
11	Uda	Tagawa	Aug 17	Sep 28	13	-
12	Ishikawa	Ishikawa	Aug 18	Aug 25	2	-
13	Kikuta	Krota	Aug 19	Oct 7	79	Ibaraki Pref.
14	Shinobu	Kamiizaka	Aug 20	Sep 19	15	Date County
15	Nishishirakawa	Tenzin	Aug 22	Oct 17	71	Tochigi Pref.
16	Iwase	Sugakawa	Aug 23	Sep 1	1	-
17	Kawanuma	Sakashita	Aug 28	Oct 4	72	Niigata Pref.
18	Hyoba	Kawamae	Aug 31	Sep 25	15	Yuha County
19	Higashishirakawa	Okada	Sep 18	Sep 26	20	-

**Table 3:** Motility and cholera diffusion routes in 1895.

**Outbreak Order**	**Infected County**	**First Infected Village**	**Entry Date**	**Termination Date**	**Cholera Cases**	**Diffusion Route**
1	Iwamae	Onahama	Jun 25	Nov 5	104	-
2	Kikuta	Izumi	Jul 1	Nov 12	54	Ibaraki Pref.
3	Nishishirakawa	Kamanoko	Jul 24	Nov 10	41	-
4	Minamiaizu	Inan	Aug 11	Aug 13	1	-
5	Iwaki	Kamiya, Kamata	Aug 12	Oct 14	50	Ibaraki Pref.
6	Higashishirakawa	Sasaya	Aug 13	Oct 23	28	-
7	Date	Yuno	Aug 16	Oct 12	105	Miyagi Pref.
8	Azumi	Koriyama	Aug 17	Oct 7	10	Yuha County
9	Tamura	OnoShinmachi	Aug 19	Oct 19	16	Iwaki County
10	Shinobu	Iizaka	Aug 22	Oct 24	80	Date County
11	Uda	Ono	Aug 29	Oct 29	16	Miyagi Pref.
12	Yuha	Kunohama	Aug 30	Oct 24	57	Iwaki County
13	Ishikawa	Aida	Sep 1	Sep 18	1	-
14	Iwase	Sukagawa	Sep 2	Nov 16	6	Shinobu County
15	Adachi	Wakizaka	Sep 7	Oct 7	10	Miyagi Pref.
16	Hyoba	Niiyama	Oct 15	Nov 11	20	-
17	Onuma	Hongo	Nov 5	Nov 20	5	-

A variety of GIS-based methods were used to digitize and align the data. All processing was performed using ARCGIS (version 9.2) software from ESRI. In order to trace the historical locations mentioned in the two reports, a digital image of the 1898 *Dai Ni-Hon Kan-Katsu-Bun Chi-Tzu *(a historical gazetteer map) was used for georeferencing. Data for the locations of infected villages and disease attributes were manually digitized and used to create two geodatabases. The 1882 version contains a time-space matrix of epidemic diffusion among 42 infected villages; the 1895 matrix covers 96. The two databases were employed to make comparisons of disease patterns and diffusion systems between 1882 and 1895. Due to improvements in sanitary systems, the 1895 report contains more detailed information (e.g., household identification) and was therefore used for diffusion modelling.

Mechanisms that influence the spread and spatial patterns of a disease or other phenomenon are at the core of diffusion studies [8]. Accounts of the spread of an infectious disease are usually reported as *relocation diffusions *or *expansion diffusions*, with the three main expansion processes being *contagious*, *hierarchical*, and *mixed *[2]. We hypothesized that the time-ordered cholera diffusion sequence in Fukushima was affected by its geographical setting, in which functional relationships between the residents of infected counties and distances from epidemic origins can be determined as a logarithmic regression model taking the form of:

*Log T*_*i *_= *β*_1 _+ *β*_2_*Log H*_*i *_+ *β*_3_*Log D*_*i *_+ *u*_*i*_,

where *Hi *is the number of households in an infected village, *Di *the direct distance (in kilometers) from the location of origin to the village where the fist cholera cases were reported, and *ui *a random disturbance; *β*_1 _is a constant. The data distribution clearly indicates the presence of outliers among *Hi*, *Di*, and *Ti*, necessitating a transformation step to create a standard distribution. The logarithmic transformation sections of equation (1) served this purpose.

In this model, the independent variables *Hi *and *Di *display a double-logarithmic relationship with *Ti *that makes it possible to represent a mix of two diffusion processes – contagious and hierarchical. *Hi *represents the hierarchical component of the spreading process and *Di *the contagious component. Accordingly, statistical significance for *Hi *is an indicator of hierarchical diffusion, and statistical significance for *Di *an indicator of contagious diffusion. Since a mixed diffusion requires statistically significant results for both *Hi *and *Di*, t-test results and *r *coefficients were used to assess correlation levels between independent variables.

## Results

The combination of GIS-based techniques and diffusion modelling allowed us to identify cholera diffusion routes and to visualize outbreak dynamics. To analyze diffusion processes we will present our results in two parts: disease pattern visualization followed by diffusion system identification.

### Visualization of Disease Patterns

**Figure 3:**
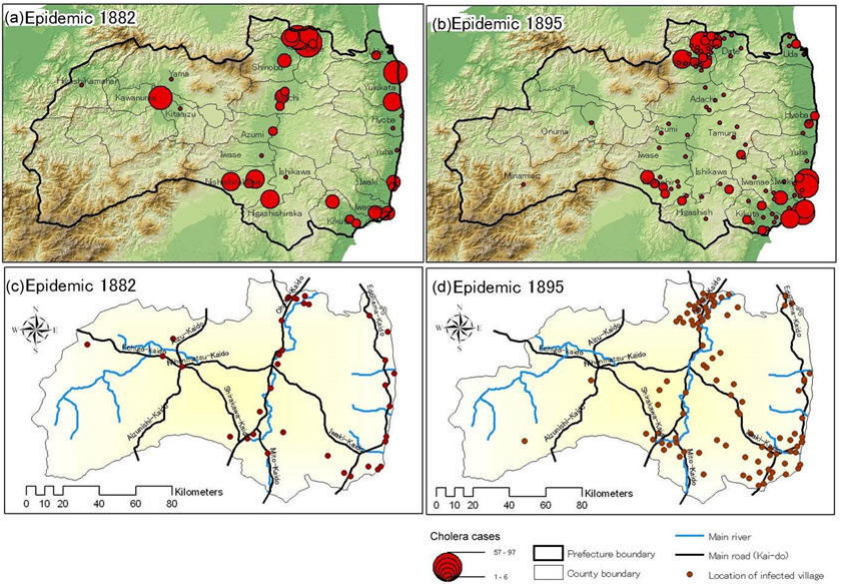
Cholera diffusion patterns in 1882 and 1895 for geographic terrain and traffic network comparisons.

An overlay map of cholera case locations and traffic networks is shown in Figure 3. Epidemic pattern changes between Fukushima villages between 1882 and 1895 are clearly evident in Figures 3a and 3b. The 1882 distribution was spatially located in the central-inland area, eastern coastline, and large valleys in the western region; the 1895 distribution heavily affected the central inland, coastal areas, and mountains in the eastern part of the prefecture. The 1895 distribution also shows concentric patterns in the north inland region and south coastal area. Figures 3c and 3d show the locations of infected villages in terms of Fukushima's main traffic networks. In 1882 the spread was limited to locations along those networks. In 1895 the number of infected villages increased, with many located more than 50 kilometres from previously identified hot spots. Features identified from figure comparisons include:

1. Geographical barriers had a greater effect on 1882 diffusion patterns than those of 1895. Village accessibility and geographical conditions may have limited the spread of cholera during the earlier outbreak.

2. Diffusion patterns seem to have been affected by boundary reforms that occurred between 1882 and 1895. Specifically, the weakened diffusion in the mountainous western region in 1895 may be attributed to the breaking up of a western county and placing part of it under the jurisdiction of Niigata prefecture.

3. Disease patterns for both outbreaks were clustered in transportation hubs, but the 1895 clusters expanded in the north-inland, south-inland, and southeast coastal areas.

### Diffusion System Identification

**Figure 4:**
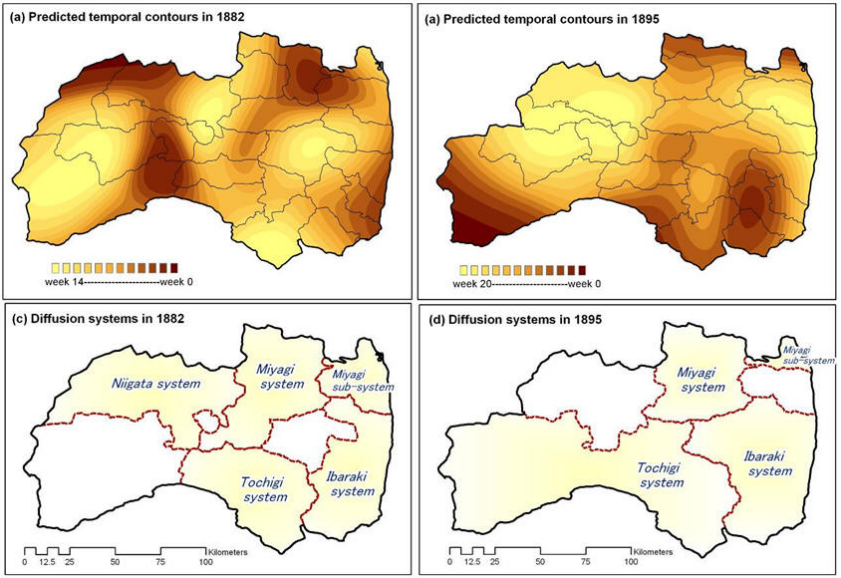
Predicted temporal contours and identified diffusion systems in 1882 and 1895.

To identify diffusion system boundaries, we applied an inverse distance weighted (IDW) interpolation technique (a function found in the spatial analysis toolbox of the ArcGIS software) to map their temporal features. Specifically, outbreak dates of index cases in each county were used to create temporal diffusion contours for 1882 and 1895. Temporal diffusion degrees were visualized as color gradations for predicting system boundaries (Figs. 4a and 4b; counties affected by specific diffusions are shown in Figs. 4c and 4d). Note that the 1882 outbreak had five diffusion systems: Niigata, Miyagi, a Miyagi subsystem, Tochigi, and Ibaraki. Coverage areas associated with each diffusion system underwent change between the two outbreaks; the Niigata system did not appear in 1895.

Fukushima faces the Pacific Ocean and shares boundaries with six other prefectures – two factors leading to increased complexity in terms of disease introduction. As shown in Figures 4c and 4d, different diffusion systems affected separate regions of the prefecture, with five origins tied to the 1882 outbreak and four to the 1895 outbreak. The first case report in 1882 came from Wakamatsu, a traffic hub in the west; in 1895 the first report came from Onahama, a fishing village on the coast. The primary diffusion points for the two outbreaks did not include either village. Instead, key entry routes have been traced to the neighbouring prefectures of Ibaraki, Miyagi, Tochigi, and Niigata. It is important to note that while textual analyses of the two cholera epidemic reports support efforts to identify possible origins, GIS-based techniques facilitate identification of the degrees to which different diffusion systems were affected by shared origins.

### 1895 Diffusion Process

**Figure 5:**
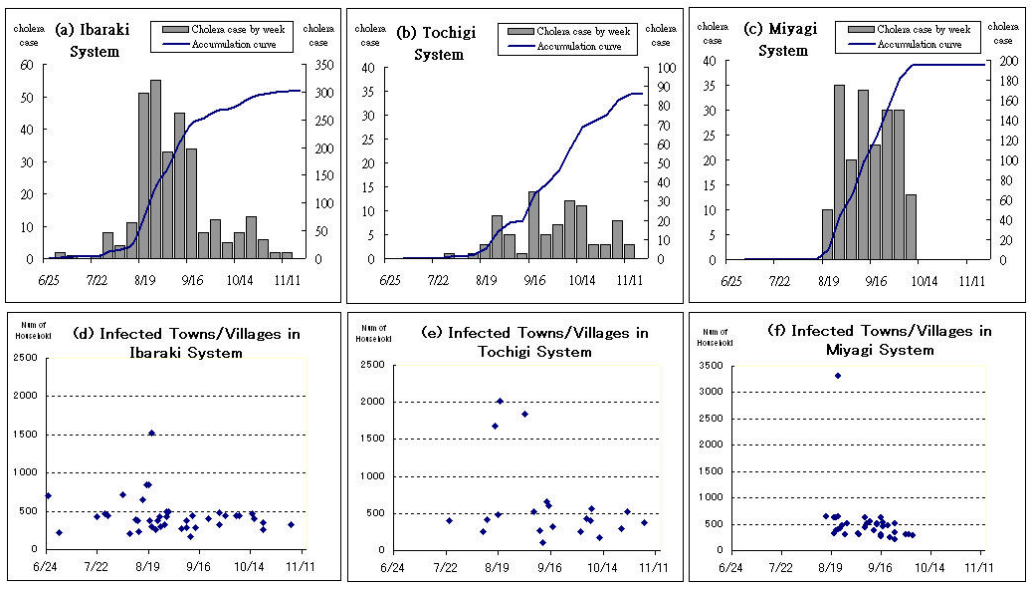
Graphs for comparing epidemic waves and numbers of infected households over identical temporal trajectories.

Once a diffusion system was identified, a geostatistical analysis was performed to determine diffusion process type. Due to integrity limitations for the 1882 data, the analysis was only applied to the 1895 diffusion. Prior to modelling each process, we compared three factors for each infected village to identify temporal and spatial diffusion dynamics: number of households, date of first case, and total number of cases. The number of households in infected villages described as susceptible represents urban diffusion systems; increases or decreases in daily accounts of cholera cases represent epidemic waves. Data for each factor were systematically compared with the temporal axes of various diffusion systems identified for the 1895 outbreak. The graphs in Figures 5a–f illustrate the waves of each diffusion system by week (left y axis) and accumulated over time (right y axis). These graphs were used for comparisons with graphic data on index case locations over the same temporal trajectory.

The Ibaraki system along the coast lasted the longest (140 days) during the 1895 outbreak (Figs. 5a and 5d). Cholera was reported in a larger village on August 21 – almost two months after the Ibaraki index case; however, the number of cases in larger villages reached their peaks at roughly the same time. For the Tochigi system, no relationship was found between village size distribution and epidemic curve over time (Figs. 5b and 5e), indicating that the index case may have occurred by chance outside of geographical influences. The Miyagi system epidemic curve represents the last and shortest Fukushima diffusion: a hierarchical pattern in which larger towns were infected in less than one week, meaning that peaks occurred very quickly (Figs. 5c and 5f).

A temporal investigation summary of infected villages/cholera cases, diffusion time period, peak of epidemic wave, date of arrival in larger towns, and type of accumulation curve is presented as Table 4. Note that even though outbreaks varied across different diffusion systems, dates of arrival in larger towns are very close to each other. In addition, the peaks of epidemic waves in the Miyagi and Ibaraki systems were temporally similar, while the Tochigi system endured several waves with irregular peak dates. Finally, accumulation curve types differed according to diffusion system: the Miyagi system had a short and rapid curve, the Ibaraki system a range of curves reflecting diverse phases, and the Tochigi system a continuing growth curve.

**Table 4:** Summary of epidemic wave features for each diffusion system.

***Diffusion System***	***Numbers of Infected Villages/Cholera Cases***	***Diffusion Time Period***	***Date of Epidemic Wave Peak***	***Date of Arrival in Major Town/Village***	***Accumulation Curve Type***
Miyagi	33/195	8/16~10/7 (52 days)	8/22, 9/21	8/23	short and rapid
Ibaraki	37/301	6/25~11/12 (140 days)	8/19	8/21	diverged into three stages
**Tochigi**	18/86	7/24~11/8 (107 days)	9/16, 10/5, 10/11	8/20	continued growth

The data were integrated into a multiple regression model for quantitative evaluation. Models of dynamic relationships between the diffusion curves and their geographic locations were individually applied for the Miyagi, Ibaraki, and Tochigi systems. In the Miyagi system, statistically significant relationships were identified between *LogTi(Time) *and both the *LogHi(Household) *and *LogDi(Distance) *variables, indicating a mixed diffusion process (Table 5). In the Ibaraki system, a statistically significant and positive relationship was noted between *LogTi(Time) *and *LogDi(Distance)*, but not between *LogTi(Time) *and *LogHi(Household)*, indicating a distance-dominated or purely contagious diffusion. No statistically significant associations were identified in the Tochigi system, meaning that it cannot be explained in terms of expansion diffusion.

**Table 5:** Results from multiple regression analysis for identifying diffusion processes.

System	n	*β*_2_	*β*_3_	*r*_*HD*_	*r*^2^	*Logβ*_1_	Process
Miyagi	33	-0.682**	0.764**	0.763	0.583	5.307**	Mixed
Ibaraki	37	0.003	0.788**	0.737	0.516	1.500	Contagious
Tochigi	18	-0.226	0.486	0.453	0.205	3.944	Unknown

**Significant at p = 0.01.

## Discussion

The cholera outbreaks that are the focus of this study occurred during a period in which sanitation concepts and initial sanitation guidelines were being promoted by the Meiji government. It is a well-studied topic, resulting in a large literature that not only focuses on the disease but also uses it as a frame for understanding societal change (see, for example, [7,11-13]). We used cholera outbreaks in Fukushima prefecture during the late nineteenth century as a frame for exploring changes in geographical structures, emphasizing the construction of geographical values for understanding regional change in modern Japan.

### Change in Geographical Structure

Data accuracy issues and uncertain boundaries often limit efforts to model historical disease diffusions. Our analysis was facilitated by rich data sources (two cholera epidemic reports) and GIS-based techniques that allowed us to digitize the locations of infected villages in order to identify regional patterns. The GIS tools also facilitated reconstructions of temporal-spatial patterns of cholera diffusion to perform comparisons in terms of geographic terrain and traffic networks.

We made three primary observations concerning the spatial characteristics of geographic structures. First, the division of Fukushima into a coastal area, inland valley, and western highlands clearly affected diffusion patterns during the 1882 outbreak. Changes in those patterns between 1882 and 1895 reflect increased accessibility to inland and coastal villages. Second, disease patterns that followed main roads or clustered around certain traffic nodes serve as indicators of population distributions and as references for analyzing economic activity in late nineteenth century Fukushima. Third, identified origin locations and diffusion routes from neighbouring prefectures can be used as evidence for determining the movement of people and goods between prefectures.

### Regional Interaction Dynamics

Whereas interactions between infected hosts and a socio-ecological environment are critical for understanding how and where infectious diseases spread, diffusion patterns provide clues to understanding regional interactions. Our results strongly support the notion that cholera diffusions in late nineteenth century Fukushima were dominated by different systems in separate regions. Accordingly, changes in the visualized boundaries of each system may represent interaction dynamics between prefectures.

A comparison of the 1882 and 1895 cholera outbreaks hint at two important changes in Fukushima. First, the significant decrease in disease diffusion in the western highlands in 1895 may be explained by a change in administrative boundaries that occurred in 1886. Specifically, part of a large county was put under the jurisdiction of Fukushima's western neighbour, Niigata prefecture (Fig. 1). The transferred region was historically referred to as *Echigo *country; its residents were more closely tied to Niigata prior to the Meiji restoration. The late nineteenth-century reform may have further reduced social and economic exchanges between the two prefectures – changes reflected by a decrease in disease diffusion. In addition, a section of the Tokyo-Sendai railway was opened in 1887; its route through central Fukushima increased interactions with and between neighbouring prefectures to the north and south. Combined with other infrastructure projects, these changes may explain the appearance of clusters of infected villages around traffic hubs in the north inland region and along the southeast coast.

### An Emerging Urban System

**Figure 6:**
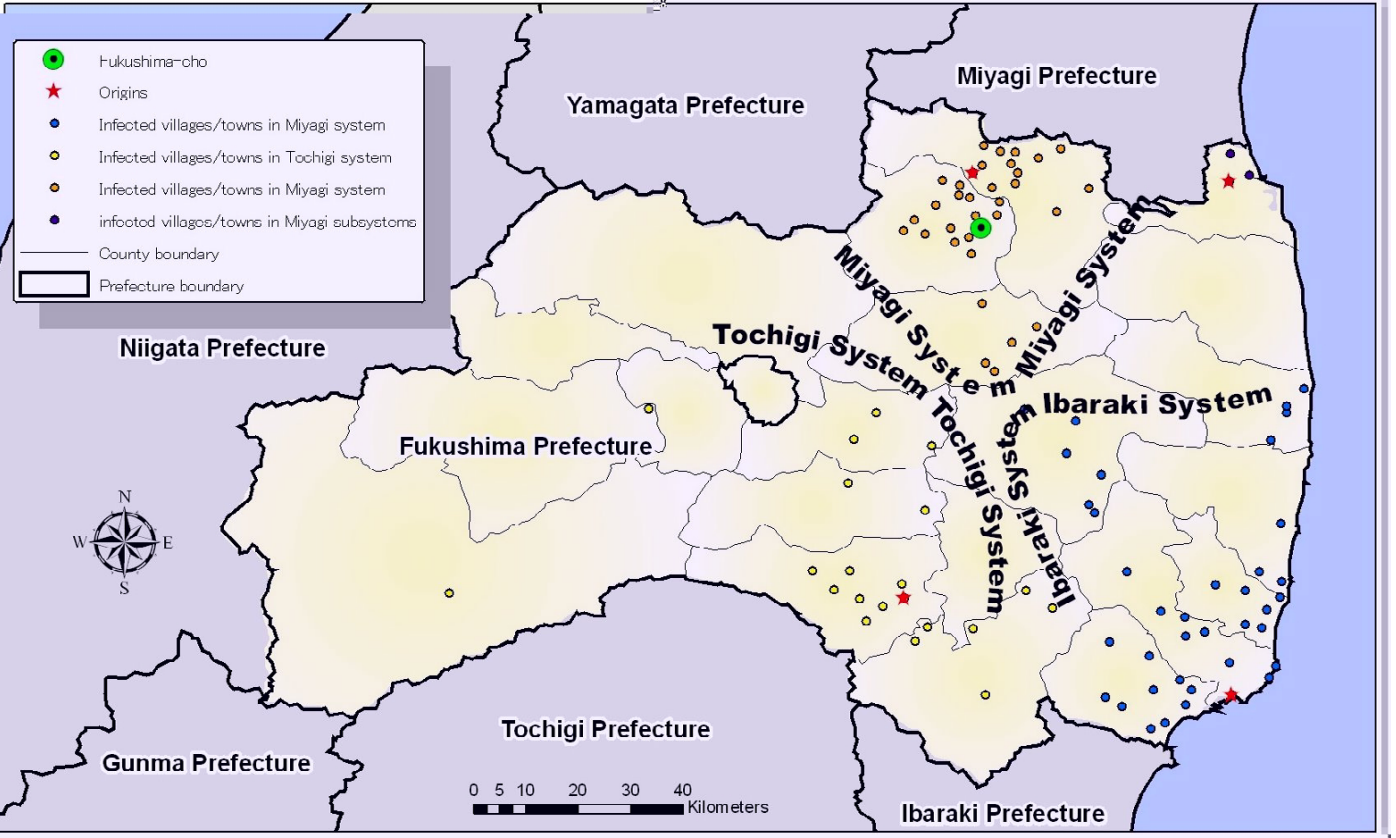
Structure of 1895 outbreak diffusion systems.

Differences in diffusion patterns can be explained by specific geographical contexts and regional interactions. In this case study, results from diffusion models shed light on the emergence of an urban system within the identified diffusion systems. Specifically, the distribution of infected villages by size may represent such an emergence during the cholera outbreaks. Our finding that the Miyagi diffusion system consisted of a mix of hierarchical and contagious processes suggests the formation of a small urban system in the northern inland area of Fukushima prefecture. Figure 6 illustrates the structure of the Miyagi diffusion system, in which Fukushima-*cho *(Fukushima City) was the major centre. Due to increased accessibility and interactions along transport networks, the larger villages and towns in the Miyagi diffusion system may have been hierarchically integrated into a larger urban system shared with its northeast neighbouring prefecture.

There is no evidence of urban systems developing in Ibaraki or Tochigi prefectures, but our data do reveal other regional features. The diffusion process in the Ibaraki system is representative of a common form of contagious diffusion found in coastal areas where fishing is the main economic activity. The importance of fishing to the Japanese diet may have further facilitated disease diffusion. The data for the Tochigi system do not support a hierarchical or contagious process in the southwest region of Fukushima. Although the Tochigi system affected a larger region than the other systems in terms of land area, only 18 villages reported infections. Due to its comparatively complex landforms (Figs. 1 and 3), a relocation diffusion process may have occurred in this region.

## Conclusion

In this paper we concentrated on the geographical dynamics of cholera diffusion in modern Japan and described a method for identifying spatial and temporal epidemic diffusion patterns, systems, and processes. Our case study of cholera outbreaks in late nineteenth century Fukushima prefecture reveals changes in geographical structure and in internal and external interactions, as well as the emergence of an urban system. We suggest that our approach can be useful for understanding both the temporal-spatial patterns of infectious diseases and the characteristics of regional change in modern Japan. We will continue to test this framework by investigating various historical diseases across different prefectures.

## Competing interests

The author(s) declare that they have no competing interests.

## Authors' contributions

CLK conceptualized the study, collected and analyzed the data, and produced the original draft. HMF participated in data collection and provided a critical review of the manuscript. All authors read and approved the final manuscript.
